# Editorial: Added value of 3D imaging in the diagnosis and prognostication of patients with right ventricular dysfunction

**DOI:** 10.3389/fcvm.2023.1356294

**Published:** 2024-01-09

**Authors:** Attila Kovács, Márton Tokodi, Elena Surkova

**Affiliations:** ^1^ Heart and Vascular Center, Semmelweis University, Budapest, Hungary; ^2^ Department of Surgical Research and Techniques, Semmelweis University, Budapest, Hungary; ^3^ Royal Brompton and Harefield Hospitals, Part of Guy’s and St. Thomas’ NHS Foundation Trust, London, United Kingdom

**Keywords:** right ventricle, right ventricular dysfunction, 3D echocardiography, cardiac magnetic resonance imaging, diagnostics, outcomes, prognostication

**Editorial on the Research Topic** Added value of 3D imaging in the diagnosis and prognostication of patients with right ventricular dysfunction

Right ventricular (RV) function is an important prognostic factor in various cardiovascular conditions, such as in heart failure with reduced and preserved left ventricular (LV) ejection fraction (EF) or in pulmonary arterial hypertension (1). The quantification of RV function is also a cornerstone of perioperative risk assessment (2) and the management of patients with mechanical circulatory support devices or adults with congenital heart disease (3). Nevertheless, the precise assessment of RV function is challenging due to its complex geometry and mechanics. Three-dimensional (3D) imaging techniques may help clinicians overcome some of these hurdles, allowing them to capture even subtle changes in RV function related to pressure- and volume overload (4), which may be undetectable using conventional imaging parameters. Thus, advanced 3D imaging-based indices of RV function may improve diagnostics and prognostication in numerous diseases (5).

This Research Topic comprises articles providing valuable insights into the intricacies of RV mechanics in both health and disease and showing the added value of 3D imaging in diagnosing RV dysfunction and predicting outcomes.

Using cardiac magnetic resonance (CMR) imaging—the gold-standard imaging modality for assessing RV size and function, a group of investigators analyzed the RV myocardial architecture in two separate articles. In the first one, Kiss et al. described the age- and sex-specific characteristics of RV compacted and trabeculated myocardium using CMR in 200 healthy volunteers. They found that RV compacted (RV-CMi) and trabeculated myocardial mass indices (RV-TMi) were higher in men than in women, and RV-TMi decreased with advancing age in the latter group. Furthermore, LV-CMi and LV-TMi, RV end-systolic volume, and sex were independent predictors of RV-TMi. In the second article, Kiss et al. measured RV volumetric, functional, and feature-tracking strain parameters in 100 patients with LV non-compaction (LVNC) phenotype and normal LVEF and compared them to those of 100 age- and sex-matched healthy controls. They observed that patients with LVNC had higher RV volumes and lower RV global and septal strain values than controls. Twenty-two percent of the analyzed LVNC patients had an RV-TMi above the reference range, and these patients had higher biventricular volumes, lower biventricular EFs, and worse RV strains than patients with normal RV-TMi. Moreover, there was a strong positive correlation between RV-TMi and LV-TMi, and both showed inverse relationships with RV function.

Besides CMR imaging, 3D echocardiography also has a crucial role in assessing RV size and function. Importantly, 3D echocardiography-derived parameters (e.g., RVEF) have significant prognostic utility, which was also demonstrated by Nabeshima et al. in 392 patients with asymptomatic aortic stenosis. They found that a lower RVEF at baseline was associated with an increased risk of cardiac events. Moreover, RVEF had incremental prognostic value over indexed aortic valve area, LVEF, and two-dimensional echocardiography-derived RV parameters.

In another study, Lan et al. investigated the effects of combined ambrisentan and phosphodiesterase type 5 inhibitor therapy on RV-pulmonary artery coupling (RV-PA coupling, assessed by different echocardiographic parameters, such as the ratio of 3D RV stroke volume and 3D RV end-systolic volume) in a retrospective study including 27 patients with severe pulmonary arterial hypertension. Six months of therapy resulted in significantly improved RV-PA coupling, World Health Organization functional class, 6-min walk distance, N-terminal pro-B-type natriuretic peptide concentration, and reduced pulmonary artery pressures and pulmonary vascular resistance assessed by right heart catheterization.

Beyond the assessment of global RV function, 3D echocardiography, in combination with advanced post-processing software solutions, can also be used for the comprehensive analysis of the RV contraction pattern. One such tool is ReVISION—a thoroughly validated, FDA-cleared, commercially available software solution—which decomposes the motion of the RV along its three orthogonal axes (i.e., longitudinal, radial, and anteroposterior axis), quantifies the contribution of these three motion components to global RV function, and computes 3D RV longitudinal, circumferential, and area strains (6, 7). ReVISION has already been used to characterize the contribution of the three motion components to global RV function in healthy adults (8, 9). However, this software solution has not yet been thoroughly tested in healthy pediatric cohorts. Motivated by this, Valle et al. initiated a two-center study to analyze the RV motion components using ReVISION in healthy children. They demonstrated that assessing the components of RV motion is also feasible in a pediatric population and found that shortening along the anteroposterior axis is the dominant component of RV contractions in healthy children.

To investigate the prognostic value of 3D RV strains measured using ReVISION, Kitano et al. sought to analyze the data of 341 patients with various cardiac diseases. 3D RV strain values (i.e., 3D RV global longitudinal, circumferential, and area strains) were significantly associated with the composite endpoint of cardiac death, ventricular tachyarrhythmia, or heart failure hospitalization, even after adjusting for age, chronic kidney disease, and LV systolic and diastolic parameters. Similarly, Tolvaj et al. also aimed to determine the prognostic power of 3D echocardiography-derived LV and RV strains. In their cohort of 357 patients with different left-sided cardiac diseases, impaired values of 3D LV and RV global circumferential strains were associated with long-term all-cause mortality, emphasizing the prognostic relevance of biventricular circumferential mechanics.

ReVISION was also tested by Evrard et al., who investigated patients ventilated due to moderate-to-severe acute respiratory distress syndrome (ARDS): 21 with ARDS related to SARS-CoV-2, 22 with ARDS unrelated to SARS-CoV-2, and 21 without ARDS. They performed a 3D transesophageal echocardiographic examination on each patient within 24 h after admission and found that RV systolic dysfunction is more pronounced in ARDS unrelated to SARS-CoV-2 than in SARS-CoV-2-related ARDS. Moreover, when analyzing the RV contraction pattern using ReVISION, they observed that the contribution of radial shortening to global RV function was reduced in patients with ARDS unrelated to SARS-CoV-2 compared to the other two groups, whereas the contributions of the other motion components (i.e., shortening along the longitudinal and anteroposterior axes) were unchanged.

Remaining in the realm of intensive care units and operating theatres, Keller et al. analyzed the associations between 3D transesophageal echocardiography-derived parameters of regional RV function and short-term outcomes in 357 patients undergoing elective cardiac surgery. They observed that a higher ratio of apical vs. inflow tract stroke volumes [assessed using their previously published custom-made software solution (10)] was independently associated with the composite of in-hospital mortality and/or the need for extracorporeal life support, underlining the importance of RV evaluation on the segmental level.

Acquiring 3D echocardiographic datasets suitable for 3D analysis might not always be feasible from the apical view. To explore whether 3D datasets can also be acquired from an alternative view, Ferraro et al. measured RV volumes on 3D echocardiographic datasets acquired from apical and subcostal views in pediatric patients and compared these measurements to the corresponding CMR-derived values. RV volumes measured from both echocardiographic views showed similarly good agreement with the CMR-derived RV volumes, confirming that the subcostal view can be an alternative to the apical view in this context.

Last but not least, in a comprehensive review article, Randazzo et al. discussed the current capabilities of 3D echocardiography to enhance RV evaluation and speculated on what the future may hold for the echocardiographic assessment of the RV.

In conclusion, this collection of articles underscores the pivotal role of 3D imaging in assessing RV structure and function across diverse clinical scenarios. Beyond providing a better understanding of the (patho)physiology of the RV, these advanced imaging techniques have true potential to enhance the detection of RV dysfunction and risk stratification, ultimately leading to improved patient care and outcomes.
